# Trends and Prospects of Low-Density Lipoprotein Cholesterol in Stroke: A Bibliometric Analysis

**DOI:** 10.7759/cureus.69492

**Published:** 2024-09-15

**Authors:** Bofeng Yang, Xianjun Ma, Li Yang, Guangrong Bian, Benyu Qiao, Hongxiang Lu, Zhuqing Wang, Tian Zhang, Ying Cheng

**Affiliations:** 1 Department of Neurology, Lianyungang Affiliated Hospital of Nanjing University of Chinese Medicine, Lianyungang, CHN; 2 Department of Acupuncture, Lianyungang Affiliated Hospital of Nanjing University of Chinese Medicine, Lianyungang, CHN; 3 Department of Laboratory Medicine, Lianyungang Affiliated Hospital of Nanjing University of Chinese Medicine, Lianyungang, CHN

**Keywords:** bibliometric analysis, low-density lipoprotein-cholesterol, review, stroke, trend

## Abstract

Management of low-density lipoprotein cholesterol (LDL-C) in stroke is a crucial component of cardiovascular disease care. Recent years have seen substantial progress in understanding and managing LDL-C in the context of stroke. This study utilized bibliometric methods to analyze and synthesize trends in this area over the past decade, incorporating 2,841 publications from the Web of Science database. The analyses included trend topic analysis, co-authorship analysis, and co-citation analysis. The findings indicate that research had predominantly concentrated on epidemiological studies related to pharmacological management strategies. Future research is expected to continue exploring lipid-lowering therapies, including both established treatments like statins and newer drugs such as proprotein convertase subtilisin-kexin type 9 inhibitors. Assessing residual cholesterol and employing Mendelian randomization techniques may become potential research hotspots. The *New England Journal of Medicine* is the most globally influential journal, while *Circulation* holds the most influence within the field, and *Atherosclerosis* ranks as the most prolific. International collaboration in this research area was strong between the USA and England, followed by the USA and China. However, collaboration between productive institutions in the USA and China remains limited, highlighting the need to strengthen partnerships between these institutions to further advance the field.

## Introduction and background

Low-density lipoprotein cholesterol (LDL-C) is implicated in the pathology of various diseases, including cardiovascular disease [1], osteoarthritis [2], diabetes [3], and cancer [4-6]. Among these, elevated LDL-C is well-established as a major risk factor in the development and progression of cardiovascular disease [7]. Stroke is a devastating cardiovascular event, and ranks as the second leading cause of mortality globally [8]. The management of LDL-C in the context of stroke presents a clinical challenge, as current clinical practice guidelines advocate for the reduction of LDL-C levels to mitigate the risk of recurrent ischemic stroke [9]. Conversely, in hemorrhagic stroke, an inverse relationship between LDL-C levels and stroke risk has been observed [10]. Despite statin therapy remaining the cornerstone of lipid-lowering treatment, with its benefits far outweighing the risks of adverse effects [11], over half of the patients fail to achieve a target LDL-C level within two years of receiving statin therapy [12].

Over the past decade, the understanding and management of LDL-C in stroke have improved. Novel pharmacotherapeutic agents, particularly proprotein convertase subtilisin-kexin type 9 (PCSK9) inhibitors, including small interfering ribonucleic acid (siRNA) and anti-PCSK9 monoclonal antibodies, significantly influenced clinical strategies for managing LDL-C levels [13]. In predicting stroke, the importance of several LDL-C-related particles has been identified, including oxidized LDL (OxLDL) and small dense LDL (sdLDL) cholesterol. Particles with diameters of 22.0-24.1 nm are classified as sdLDL [14]. Compared with overall LDL-C, sdLDL levels can predict ischemic stroke with less influence from statins [15,16]. OxLDL, formed when LDL particles are modified by oxidants, has recently been shown to predict outcomes after stroke [17].

Despite numerous studies, the specific mechanisms and management of LDL-C in stroke remain suboptimal. Summarizing current research trends and hotspots will facilitate future research. Bibliometric analysis allows for the systematic summarization and quantitative analysis of research in a given field without limiting the type of research [18]. Researchers and clinicians can use these results to conveniently understand current research hotspots and trends and to select directions for future studies.

## Review

Data collection and analysis

This study included publications concerning both LDL-C and stroke from 2014 to 2023. The search was conducted using the Web of Science Core Collection database with the strategy listed in Table 1.

**Table 1:** Search strategy

No.	Strategy
1	TS = (beta-lipoprotein Cholesterol) OR (beta lipoprotein Cholesterol) OR (low density lipoprotein cholesterol) OR (LDL cholesterol) OR (LDL cholesteryl linoleate)
2	TS = (stroke) OR (strokes) OR (apoplexy) OR (brain infarction) OR (cerebral infarction) OR (infarction encephalopathy) OR (intracranial embolism)
3	TS = (stroke volume) OR (heat stroke)
4	FPY = 2014-2023
5	(#1 AND #2 AND #4) NOT #3

Only English-language publications were included. Retracted publications or corrections were excluded. Keywords provided by authors underwent trend topic analysis. Co-authorship analysis was conducted on the top 10 countries/regions and institutions with the most publications included. Co-citation analysis was conducted on the top 30 journals most cited by the included publications. VOSviewer software (version 1.6.19; Centre for Science and Technology Studies, Leiden, The Netherlands) and the Bibliometrix package of R software (version 4.3.0; R Foundation for Statistical Computing, Vienna, Austria) were used for result visualization.

Basic characteristics

**Figure 1:**
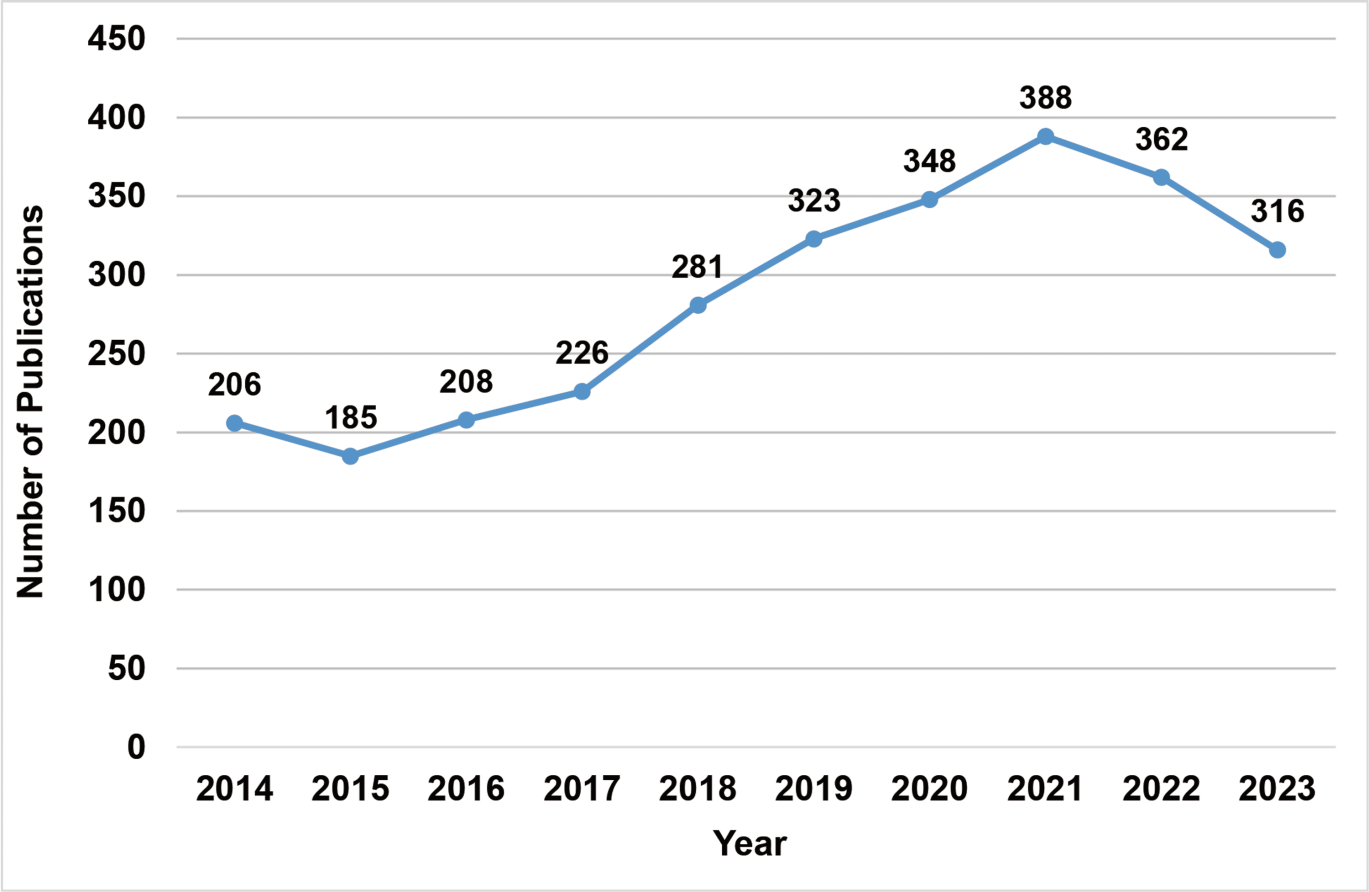
Number of annual publications

The search in the WOS database yielded 2889 publications. Among them, 42 non-English publications and six retracted publications were excluded. A total of 2841 publications were included, containing 2382 research articles, 393 review articles, and 66 publications of other types. As shown in Figure 1, annual publications increased between 2015 and 2021 but declined between 2021 and 2023. In 2021, 288 publications were the highest number published in the past decade. Table 2 shows the top 10 journals, countries/regions, and funding agencies with the most publications. *Atherosclerosis* published the most publications (n = 87). Among the top 10 productive journals, six were cardiovascular journals, two were multidisciplinary journals, one was a biochemical journal, and one was a general medical journal. China and the USA were the most productive countries, with 898 (31.59%) and 825 (29.02%) publications. The National Natural Science Foundation of China funded the most research, followed by three USA non-profit institutions. Of the top 10 funding agencies, four were biomedical companies.

**Table 2:** The top 10 journals, countries/regions, or funding agencies with the most publications

Category		Count (%)
Journal	Atherosclerosis	87 (3.06)
	PLoS One	59 (2.08)
	Circulation	58 (2.04)
	Journal of the American Heart Association	54 (1.90)
	Lipids in Health and Disease	53 (1.86)
	Journal of Stroke Cerebrovascular Diseases	52 (1.83)
	Frontiers in Cardiovascular Medicine	49 (1.72)
	Scientific Reports	49 (1.72)
	Stroke	49 (1.72)
	Medicine	46 (1.62)
Country/Region	China	898 (31.59)
	USA	825 (29.02)
	England	313 (11.01)
	Japan	209 (7.35)
	South Korea	164 (5.77)
	Canada	155 (5.45)
	Australia	150 (5.28)
	Germany	137 (4.82)
	Netherlands	131 (4.61)
	Italy	130 (4.57)
Funding agency	National Natural Science Foundation of China	267 (9.39)
	United States Department of Health Human Services	251 (8.83)
	National Institutes of Health (USA)	240 (8.44)
	National Heart Lung Blood Institute (USA)	102 (3.59)
	Amgen Company	93 (3.27)
	AstraZeneca Company	76 (2.67)
	Pfizer Company	74 (2.60)
	UK Research and Innovation	64 (2.25)
	Medical Research Council (UK)	61 (2.15)
	Merck Company	61 (2.15)

Hotspots and trends

The 30 most frequently mentioned keywords in the publications are listed in Table 3. In addition to stroke, LDL-C, and their derivative words, pathological features including atherosclerosis, inflammation, blood pressure, and triglycerides were frequently co-concerned. Atherosclerosis, often resulting from prolonged LDL-C exposure, is a primary driver of ischemic stroke [19]. In a synergistic way, LDL-C, inflammation, blood pressure, and triglycerides all influence the occurrence of atherosclerosis [1]. Co-concerned diseases or symptoms included myocardial infarction, coronary artery disease, diabetes, and acute coronary syndrome. These cardiovascular diseases, similar to stroke, are caused by atherosclerosis and are affected by LDL-C levels [1]. The incidence of diabetes is one reason that prompted investigations into alternative lipid-lowering agents beyond statins. Intensive-dose statin therapy can better reduce LDL-C levels but increase the risk of new-onset diabetes, a risk factor for stroke [20]. In terms of pharmacotherapy, statins and ezetimibe were focal points. Ezetimibe is a non-statin drug that reduces intestinal cholesterol absorption, which can be used in combination with statins to further reduce the risk of ischemic stroke [21]. Other keywords were mainly related to epidemiological aspects, including risk factors, mortality, meta-analysis, and prognosis.

**Table 3:** The top 30 keywords with the most frequencies

Keyword	Count
Stroke	372
Cardiovascular disease	276
Atherosclerosis	233
Cholesterol	191
Ischemic stroke	167
Risk factors	143
Low-density lipoprotein cholesterol	112
Lipids	111
Statin	105
Statins	104
Myocardial infarction	103
Dyslipidemia	102
Coronary artery disease	101
Epidemiology	88
Cardiovascular diseases	87
Inflammation	87
Secondary prevention	84
Coronary heart disease	83
Mortality	78
Hypertension	71
Diabetes mellitus	70
Ezetimibe	67
Meta-analysis	67
Diabetes	64
Metabolic syndrome	61
Acute coronary syndrome	55
Acute ischemic stroke	55
Prognosis	53
Blood pressure	52
Triglycerides	50

**Figure 2:**
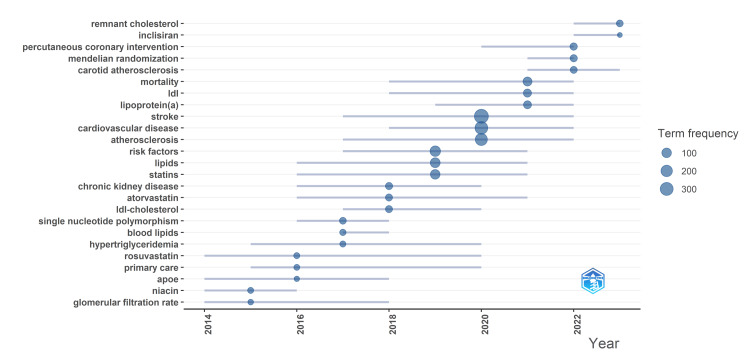
Trend topic analysis The blue line indicates the duration of the keyword’s status as a hot topic; the size of the blue dot indicates its frequency.

Figure 2 illustrates the trend of research topics with two to three keywords each year. Besides stroke, LDL-C, and their derivatives, six keywords, atherosclerosis, statins, atorvastatin, hypertriglyceridemia, rosuvastatin, and primary care, emerged as enduring research hotspots over more than five years. The sustained focus on statins as a topic of interest underscores the significant attention given to their clinical applications. Recently emerged keywords including remnant cholesterol, inclisiran, and Mendelian randomization suggest potential future focal points in the field. Remnant cholesterol, representing cholesterol fractions excluding LDL-C and high-density lipoprotein cholesterol (HDL-C) (also termed triglyceride-rich lipoprotein cholesterol), has garnered attention due to its association with residual cardiovascular risk [22]. Inclisiran is a first-in-class siRNA targeting PCSK9 approved by FDA in 2021. Phase III clinical trials have demonstrated its capacity to reduce LDL-C levels by approximately 50% [23]. Compared to anti-PCSK9 monoclonal antibodies, inclisiran offers a more durable effect with comparable safety profiles [23,24]. Mendelian randomization uses genetic variants to examine correlations between risk factors and outcomes. In cardiovascular disease, studies using Mendelian randomization have made progress, including confirming an association between low LDL-C levels and hemorrhagic stroke [25]. This methodology offers advantages over traditional clinical trials by utilizing existing data, thereby conserving resources [26].

Collaboration

**Figure 3:**
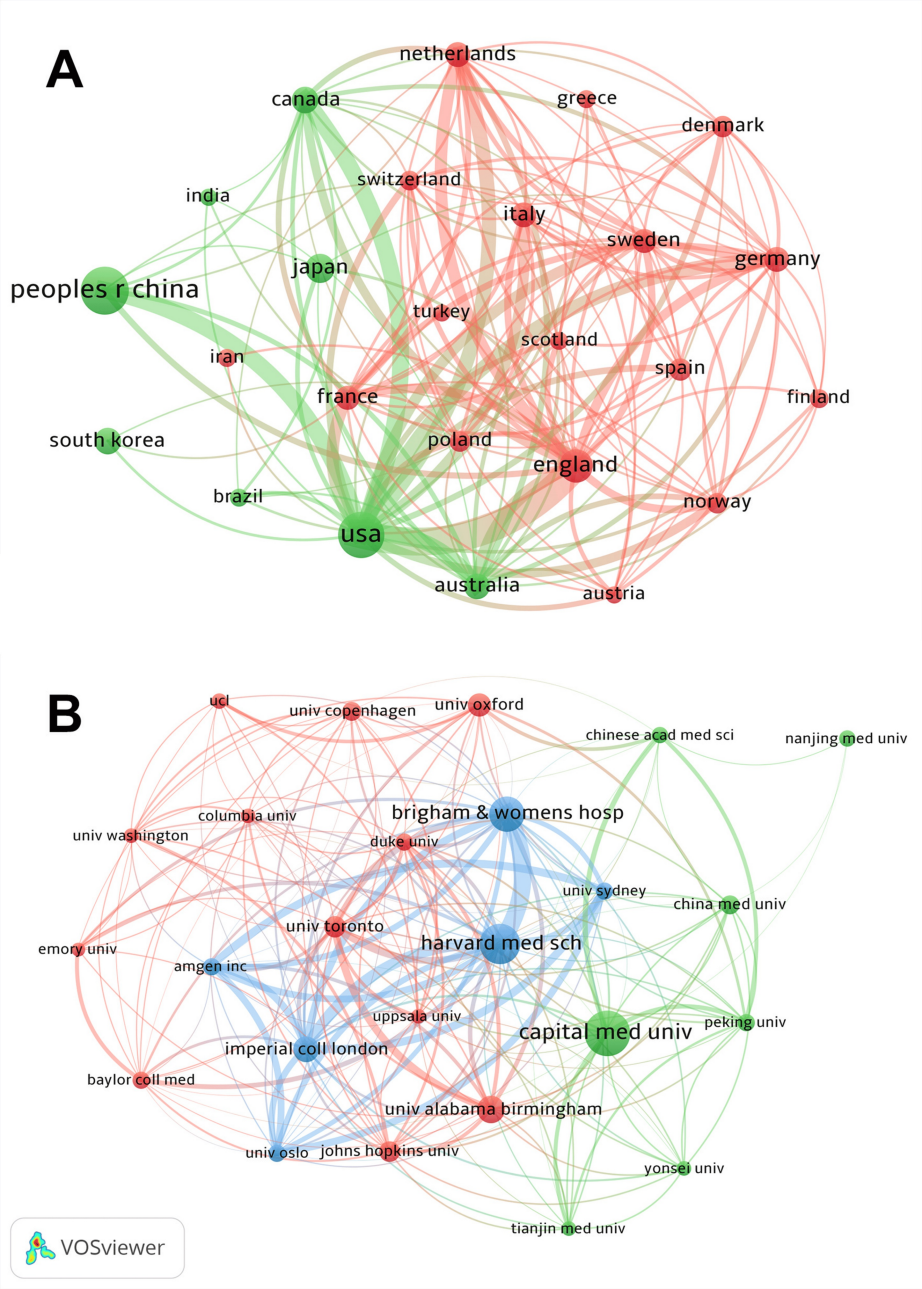
Collaboration analysis: (A) top 25 countries/regions (B) and top 25 institutions The size of the dot indicates the frequency, and the thickness of the line indicates the frequency of co-occurrence.

Analyzing international collaborations (Figure 3A) revealed two distinct clusters. Despite the most substantial collaboration being between the USA and England, these two countries did not belong to the same cluster. Interestingly, the red cluster mainly comprised European countries, while the green cluster included nations outside Europe. This suggests a propensity for European researchers to collaborate within the continent, whereas researchers from non-European countries tended to engage with peers from other non-European regions. The second largest international cooperation was between China and the USA. However, institutional collaborations between these two countries, as depicted in Figure 3B, did not exhibit strong ties. This disparity may be attributed to Figure 3B highlighting only the top 25 most productive institutions, indicating that high-level collaborations between leading institutions in China and the USA are limited, with more interactions occurring among other institutions. This observation underscores potential opportunities for enhanced collaboration between productive institutions in both countries. In Figure 3B, institutions within the blue cluster were the most closely connected ones. The collaborations among Harvard Medical School, its affiliated hospitals, the University of Sydney, Imperial College London, the University of Oslo, and the Amgen company were relatively close.

Citation preference

**Figure 4:**
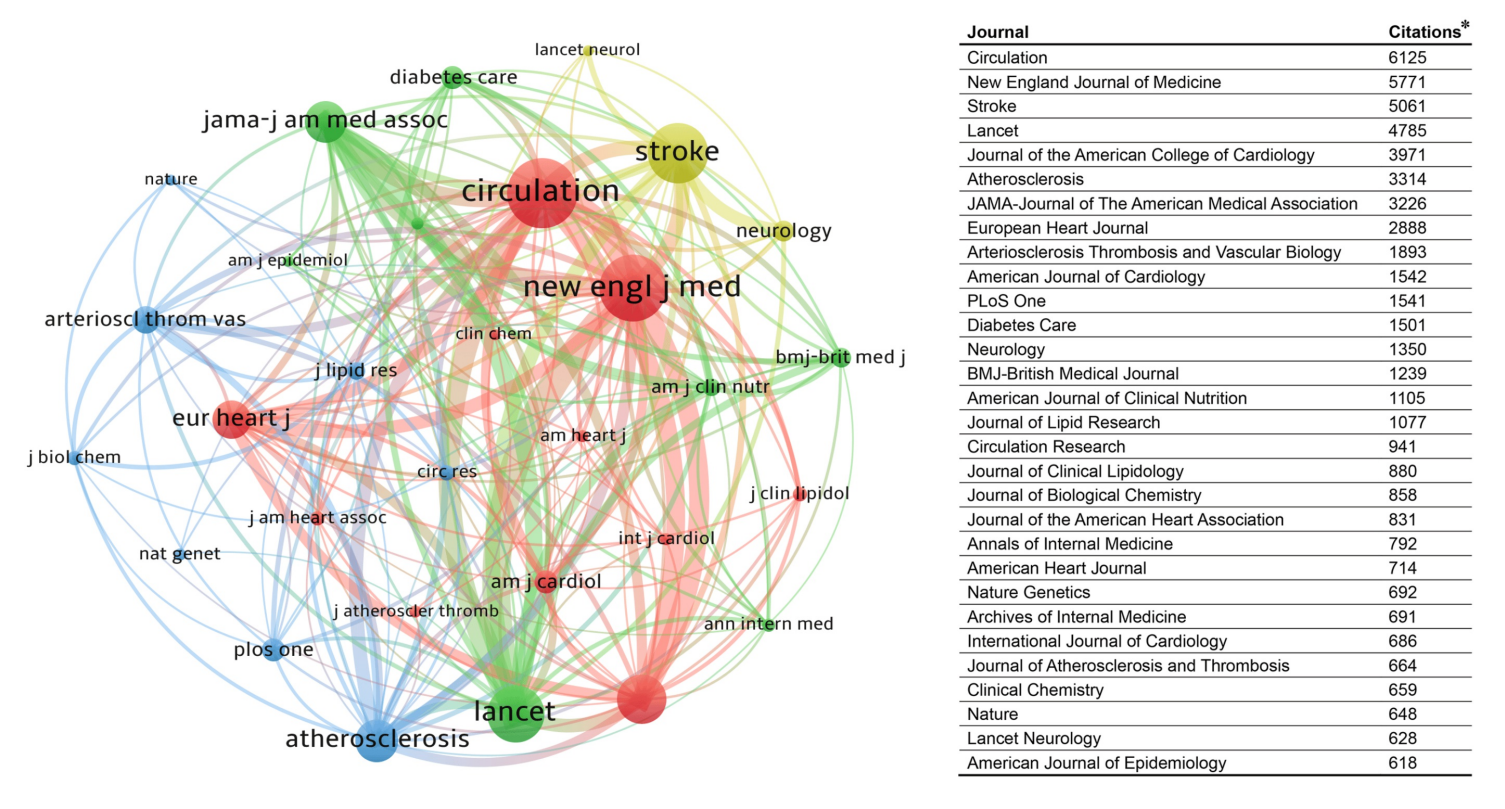
Co-cited analysis of the top 30 local cited journals ^*^Citations recorded from the included publications. The size of the dot indicates the frequency, and the thickness of the line indicates the frequency of co-occurrence.

Figure 4 shows the citation preferences within the field of LCD-C in stroke. Each color represents a cluster of frequently co-cited journals. The biggest cluster (red) contains 11 journals, mostly cardiovascular journals, characterized by strong internal correlations. Circulation is the most influential journal within the field, with the most citation records in the included publications (6125 times). Other local influential journals include the *New England Journal of Medicine*, *Stroke*,* Lancet*, *Journal of the American College of Cardiology*, *Atherosclerosis,* and *JAMA*, each cited by the included publications more than 3000 times.

Broadening the perspective to the entire scientific domain, the topic of LDL-C in stroke maintains substantial interest. Table 4 lists the top 10 publications with the highest citation counts in the WOS database. The study titled “Evolocumab and Clinical Outcomes in Patients with Cardiovascular Disease” got the most citations (3683 times, recorded on 28 July 2024). The composition of these highly cited publications suggested the emphasis on clinical pharmacotherapy for LDL-C management in stroke, with seven publications from the *New England Journal of Medicine* detailing randomized controlled trials on therapeutic efficacy and safety, and three being reviews. When assessing global citations by journals (Table 5), the *New England Journal of Medicine* held a commanding lead with 19,893 citations, followed by *Circulation*, *Lancet,* and *Journal of the American College of Cardiology*. The dominance of the *New England Journal of Medicine* in both top-cited publications and overall journal citations reflects the profound impact of studies published within its pages across the broader scientific community.

**Table 4:** The top 10 publications with the most global citations RCT, randomized controlled trial. ^a^Based on the citations recorded in Web of Science Core Collection on 28 July 2024.

Title	Publication type	Corresponding author	Journal	Publication year	Citations^a^
Evolocumab and Clinical Outcomes in Patients With Cardiovascular Disease	RCT	Sabatine MS	New England Journal of Medicine	2017	3,683
Ezetimibe Added to Statin Therapy After Acute Coronary Syndromes	RCT	Cannon CP	New England Journal of Medicine	2015	2,962
Alirocumab and Cardiovascular Outcomes After Acute Coronary Syndrome	RCT	Schwartz GG	New England Journal of Medicine	2018	1,998
Cardiovascular Risk Reduction With Icosapent Ethyl for Hypertriglyceridemia	RCT	Bhatt DL	New England Journal of Medicine	2019	1,933
Atherosclerosis	Review	Libby P	Nature Reviews Disease Primers	2019	1,608
Efficacy and Safety of Alirocumab in Reducing Lipids and Cardiovascular Events	RCT	Robinson JG	New England Journal of Medicine	2015	1,577
Efficacy and Safety of Evolocumab in Reducing Lipids and Cardiovascular Events	RCT	Sabatine MS	New England Journal of Medicine	2015	1,227
Exercise as Medicine - Evidence for Prescribing Exercise as Therapy in 26 Different Chronic Diseases	Review	Pedersen BK	Scandinavian Journal of Medicine & Science in Sports	2015	1,216
Effects of Extended-Release Niacin With Laropiprant in High-Risk Patients	RCT	Armitage J	New England Journal of Medicine	2014	1,201
Interpretation of the Evidence for the Efficacy and Safety of Statin Therapy	Review	Collins R	Lancet	2016	1,159

**Table 5:** Top 10 journals by global citations of included publications ^a^Based on the citations recorded in Web of Science Core Collection on 28 July 2024.

Journal	Citations^a^
New England Journal of Medicine	19,893
Circulation	5737
Lancet	4174
Journal of the American College of Cardiology	3531
JAMA-Journal of The American Medical Association	2830
Atherosclerosis	2652
European Heart Journal	2524
Stroke	2112
Circulation Research	1764
Lancet Diabetes & Endocrinology	1638

Considering the citation preferences and keywords listed in Table 3, mechanistic studies appear underrepresented. Such studies were absent among the highly cited publications, and related keywords seldom surfaced. The aforementioned highly cited journals mainly publish clinical and epidemiological studies; for example, the *New England Journal of Medicine* publishes almost no mechanistic studies. The heightened focus on clinical studies may be attributed to the limitations of current medications for managing LDL-C levels in stroke. Statins have long been used as a first-line drug to reduce the risk of stroke by lowering LDL-C. At maximum doses, statin therapy can reduce LDL-C levels by 55-60% [27]. Despite the benefits of statin therapy far outweighing the risk of adverse effects, the residual risk of stroke and the increased risk of new-onset diabetes mellitus and hemorrhagic stroke could not be denied [11,20]. Some efforts have been put into drug combinations to further reduce the risk of stroke on top of the LDL-C reduction achieved by statin therapy but failed [28,29]. Recently, PCSK9 inhibitors showed great potential to further reduce the LDL-C levels thus reducing the risk of stroke when added to statin therapy [30]. Some of these drugs have entered the market, with many others under investigation [13]. In reducing residual risks beyond LDL-C, recent studies revealed the potential of highly purified eicosapentaenoic acid in assisting LDL-C-lowering therapy to reduce triglyceride levels [31]. Future research is anticipated to continue emphasizing clinical studies, focusing on the long-term efficacy, safety, and subgroup-specific benefits of these novel therapeutic agents.

Limitations

This study has several limitations that warrant consideration. First, the reliance on the WOS database may introduce selection bias, as WOS does not encompass all relevant publications. Nevertheless, the exclusive use of the WOS database enhances the interpretability of the results by maintaining a consistent dataset. Merging multiple databases for keyword analysis, as discussed in a previous study, can introduce confusion and reduce clarity [32]. Secondly, while this study provides an overview of research hotspots and trends over the past decade based on publication characteristics, it does not necessarily capture the full importance of these topics. Some valuable research areas may not be reflected in the identified hotspots, suggesting that there may be significant topics that require further exploration beyond those highlighted in the current analysis. Researchers should remain vigilant in identifying and investigating emerging areas that may not yet be widely recognized.

## Conclusions

Over the past decade, research on LDL-C in the context of stroke has predominantly focused on epidemiological studies examining pharmacological management strategies to prevent stroke. Future studies are likely to continue emphasizing the clinical application of lipid-lowering therapies, including classical statin therapy, and exploring the long-term efficacy, safety, and subgroup benefits of emerging drugs such as PCSK9 inhibitors. Potential research hotspots include the assessment of residual cholesterol and the application of Mendelian randomization. The *New England Journal of Medicine* is the most influential journal, which had a large preponderance of highly cited publications and received the most global citations. *Circulation* is the most influential journal within the field, which received the most local citations. In addition, *Atherosclerosis* is the most productive journal. International collaboration, particularly between the USA and England, was prevalent in this research area, followed by cooperation between the USA and China. However, the collaboration between highly productive institutions in the USA and China remains limited, highlighting the potential and importance of strengthening partnerships between these two leading research countries to advance the field further.
